# Transferrin predicts trimethylamine-N-oxide levels and is a potential biomarker of cardiovascular disease

**DOI:** 10.1186/s12872-022-02644-3

**Published:** 2022-05-10

**Authors:** Lamuel D. Bean, Jeffrey J. Wing, Randall E. Harris, Suzanne M. Smart, Subha V. Raman, M. Wesley Milks

**Affiliations:** 1 Division of Epidemiology, College of Public Health, The Ohio State University, Columbus, OH USA; 2 Davis Heart and Lung Research Institute, College of Medicine, The Ohio State University, Columbus, OH USA; 3 Krannert Cardiovascular Research Center, Indiana University School of Medicine, Indianapolis, IN USA; 4 Division of Cardiovascular Medicine, Department of Internal Medicine, The Ohio State University, Wexner Medical Center, 473 W 12th Ave Suite 200, Columbus, OH 43210 USA

**Keywords:** Trimethylamine-N-oxide, Biomarkers, Cardiovascular disease, Cardiology, Epidemiology

## Abstract

**Introduction:**

Trimethylamine-N-oxide (TMAO) is a circulating biomarker associated with cardiovascular disease (CVD). Production of TMAO is facilitated by gut microbiota and dependent on micronutrients such as choline, betaine, and L-carnitine, present in foods such as red meat and eggs.

**Hypothesis:**

We sought to predict serum TMAO quartile levels among healthy individuals at increased risk of CVD using clinical data via an ordinal logistic model.

**Methods:**

Data from participants (n = 127) enrolled in a longitudinal observational study on CVD were used to build a predictive model for TMAO using ordinal logistic regression with demographic variables and 40 other variables considered related to CVD risk. First, univariate models for each covariate were tested (with serum TMAO quartiles as the dependent variable), and only variables with *P* < 0.30 were evaluated further. Second, demographic variables (age, gender, white vs. non-white race) were included in a multivariable model with each previously identified independent variable controlling for potential confounding. Last, the final model included fixed demographics and candidates from the confounder-adjusted model with *P* < 0.10.

**Results:**

Eight candidate variables were included in the final model, with only transferrin, high-density lipoprotein cholesterol (HDL-C) and race (white vs. non-white) showing significant associations with TMAO. Participants had 0.16 (Q2), 0.31 (Q3), and 0.20 (Q4) odds of being in a higher TMAO quartile compared with participants in the lowest transferrin quartile. Non-white participants had 2.92 times higher odds of being in the highest TMAO quartile compared to white individuals. Participants in the second quartile of HDL-C had 2.68 times higher odds of being in a higher TMAO quartile compared with participants in the lowest HDL-C quartile.

**Conclusions:**

Transferrin demonstrated a significant predictive association with TMAO and may represent a novel potential biomarker of increased CVD risk worthy of further study. These results warrant further examination of iron, metabolism, homeostasis, and gut microbiome to better understand and mitigate known increased CVD risk.

**Supplementary Information:**

The online version contains supplementary material available at 10.1186/s12872-022-02644-3.

## Introduction

Heart disease remains the leading cause of death, responsible for more than 655,000 deaths in the U.S. and 17.9 million deaths worldwide each year. Between 2016 and 2017, heart disease resulted in approximately $363 billion in costs of health care services, medicines, and lost productivity due to death in the United States [1]. Effective control of risk factors significantly reduces CVD risk and associated health care costs. Multiple factors have been identified in previous studies that elevate risk, yet data for novel factors continues to emerge.

Traditional factors used to predict atherosclerotic cardiovascular disease (ASCVD) risk and reduce costs/outcomes via treatment (e.g. with statins) include blood pressure, glucose, lipids, weight, physical inactivity, stress, smoking, and certain dietary patterns [2]. Conversely, effective control of these factors significantly reduces CVD risk and associated health care costs. Unhealthy dietary factors constitute an important component of the CVD risk factor profile, and elucidation of potential dietary biomarkers of CVD risk is an important research objective. Recent studies have revealed trimethylamine-N-oxide (TMAO) as a novel ASCVD biomarker. Gut microbiota metabolize the micronutrients choline, betaine, and L-carnitine to form trimethylamine (TMA), which is absorbed and then further oxidized into TMAO by flavin-dependent monooxygenase enzymes in the liver [3]. Past studies have found that higher TMAO serum levels are associated with increased risk of CVD [4], atherosclerosis [5], coronary artery disease [6], as well as other chronic diseases that are associated with CVD such as diabetes mellitus (DM) [7] and chronic kidney disease [8]. The growing body of evidence elucidating the mechanisms of TMAO-induced atherogenesis have basis in high-quality animal studies, one among them noting that supplementation of *Apoe*^−/−^ mice with L-carnitine leads to aortic atherosclerosis in the absence of adverse alterations in lipoprotein or glucose levels, and TMAO inhibits reverse cholesterol transport [9]. In addition, TMAO has been associated with increased platelet reactivity, such as in a study of healthy volunteers who were given choline bitartrate supplementation, which resulted in both higher TMAO levels and platelet responsiveness [10] Interestingly, an experimental diet involving consumption of 4 hardboiled eggs daily did not produce the same effect, despite having similar choline content, potentially implicating additional complexities in food matrix composition.

Dietary studies have provided consistent evidence that omnivorous diets are associated with high TMAO levels compared to vegetarian diets. Since TMAO is a metabolite of the precursor nutrients choline and L-carnitine, many studies have investigated the association between diet and TMAO levels, particularly diets high in red meats [11] or eggs; generally, omnivorous [12] and traditional Western dietary patterns rather than vegetarian ones tend to be abundant in such TMAO precursors [13]. One study found that individuals who consumed primarily omnivorous diets had a tenfold higher odds of being in a high TMAO producer group compared to individuals who consumed vegetarian diets [14].

Thus, TMAO levels demonstrate a relationship with omnivorous diets and CVD risk; however, very few studies have described other CVD-related non-dietary biomarkers associated with TMAO. The objective of this study is to explore potentially novel dietary factors or biomarkers that associate with TMAO levels, among participants at increased risk of incident ASCVD.

## Methods

Data used in this analysis were initially collected from a longitudinal observational study of 127 recruited adults greater than 40 years of age with risk factors for, but without known, ASCVD. Adults without evident ASCVD but with 2 or more risk factors (total cholesterol ≥ 240 mg/dl, systolic blood pressure ≥ 140 mmHg, diastolic blood pressure ≥ 90 mmHg, current smoking, or DM) were prospectively identified primarily by recruitment from ambulatory clinics associated with the Ohio State University Wexner Medical Center. Other participants were enrolled via flyer advertisements, word of mouth, and by visiting other clinics to recruit. No participants had a known history of iron overload or significant blood transfusion. All subjects gave written informed consent to participate in this Institutional Review Board-approved study.

All biometric measurements were obtained in person at baseline and at 2 years follow-up. Only baseline data were used for the current study due to the lack of significance in temporality and to maximize precision of the sample of data used for analysis. The leading author of this manuscript had full access to all data in this study and takes responsibility for its integrity and the data analysis.

### Outcome classification

Serum concentration of TMAO (the outcome variable) was measured with liquid chromatography-tandem mass spectrometry (LC-MS/MS). Separation for HPLC was performed on a Dionex UltiMate 3000 RSLCnano System with a Waters ACQUITY UPLC BEH amide column at 30 °C with a gradient including solvent A (NH_4_COOH (5 mM) with 0.1% formic acid) and solvent B (acetonitrile). Using the multi reaction monitoring (MRM) technique on a Thermo TSQ Quantiva (triple quadrupole MS/MS) instrument, the ion pairs of 76.1/58.1 and 85.1/66.1 were used to monitor the concentration of TMAO and the internal standard (IS) d9-TMAO, respectively. All mass spectrometer parameters were tuned to give the optimized MRM signal. For standard curve preparation for calibration, to 160 µL of MeOH, 20 µL of TMAO stock solution (at the conc. of 0.005, 0.01, 0.02, 0.05, 0.10, 0.20, 0.50, 1.0, 2.0, 5.0, and 10.0 ppm in H_2_O) were added together with 20 µL of IS at 1 µg/mL in ACN to yield standard samples (at 0.5, 1.0, 2.0, 5.0, 10, 20, 50, 100, 200, 500, and 1000 ng/mL [ppb]). The quality controls (QCs) were at 10, 100, and 500 ng/mL. For human plasma sample preparation, to 50 µL of the sample, 50 µL of IS at 1 µg/mL in ACN and 400 µL of cold MeOH (pre-chilled at − 20 °C) were added. The mixture was vortexed for approximately 3 s, kept at − 20 °C for 30 min, and centrifuged at 13,000 rpm/4 °C for 10 min. A supernatant quantity of 100 µL was transferred to glass vials for LC-MS/MS injection and measurement.

### Exposures/covariates

Demographic characteristics collected included race, gender, age, and race. History of hypertension, statin use, smoking, diabetes status, height, weight, hip and waist circumference, waist hip ratio, and body-mass index (BMI) were abstracted from medical records and collected in person. Biomarkers studied included blood pressure, Modification of Diet in Renal Disease-derived glomerular filtration rate (MDRDGFR), hematocrit, transferrin, ferritin, hepcidin, adiponectin, TMAO, blood total cholesterol, high-density lipoprotein cholesterol (HDL-C), low-density lipoprotein cholesterol (LDL-C), oxidized LDL-C, triglycerides, iron, iron saturation, ferritin, and creatinine levels [15, 16]. Baseline values of these biomarkers were used for this analysis.

### Data analysis

Participant characteristics were summarized overall to evaluate their distributions. Distributional features of TMAO and other variables were examined by standard statistical measures (means, standard deviations, and medians). For the original data set a missing outcome deletion approach was used to deal with the missingness of the outcome data. Therefore, any observations that had missing baseline TMAO data were removed. Continuous measures that were highly skewed including serum TMAO (dependent variable) were recoded into quartiles. Race was operationalized as white versus non-white based on small counts of participants of non-white races. After cleaning and managing the data set, ordinal logistic regression was used to build a predictive model for serum TMAO levels. TMAO levels were stratified into quartiles for testing associations with demographic factors (age, gender and race were fixed in the model) and 40 clinical variables potentially related to CVD risk. Initially, univariate models were tested and only covariates with *P* < 0.30 retained for further testing. Successive stages of testing were based on a stricter threshold (*P* < 0.20) in multivariable models with age, gender, and race to adjust for potential confounders. In the final model, only covariates approaching statistical significance at *P* < 0.10 were considered of value for TMAO prediction. All analyses were conducted using Intercooled Stata Version 14.1 (Stata Corp, College Station, TX) software (See Additional File 1 for dataset used for analysis).

## Results

### Clinical baseline characteristics

A total of 127 participants with reported baseline TMAO serum level data were enrolled (Table 1). On average, participants were 50 years of age (SD: 3.7), weighed 206 lbs (SD: 51.9) and had a body mass index (BMI) of 32 (SD: 7.7). The majority were white (80.3%), female (60.6%), obese (60%), and had hypertension (66.4%); 32.3% had diabetes and 36.2% were statin users. Most had never smoked (79.5%) and those who smoked (19.7%) quit more than 6 months before the study; only 1 participant was a current smoker.

**Table 1:** Characteristics of 127 participants based on available serum TMAO data

	N	% Or mean (SD)
Gender		
Female	77	60.63
Male	50	39.37
Race		
White	102	80.31
Non-White	25	19.69
Hypertension		
Yes	84	66.14
No	43	33.86
Diabetes		
Yes	41	32.28
No	86	67.72
Statin user		
Yes	46	36.22
No	81	63.78
Smoker		
Never	101	79.53
Quit > 6 Months	25	19.69
Current	1	0.79
Age (years)	127	49.40 (3.72)
Weight (lbs)	127	205.90 (51.93)
BMI	127	32.06 (7.68)
Waist (Inches)	118	39.53 (6.20)
Hip (Inches)	118	44.13 (5.91)
Waist-hip ratio	118	0.90 (0.07)
Systolic BP	127	129.44 (14.89)
Diastolic BP	127	83.64 (9.80)
Creatinine^†^	127	0.85 (0.19)
MDRDGFR*	127	86.11 (16.37)
Total Cholesterol^†^	127	196.28 (48.71)
HDL^†^	127	49.65 (15.80)
LDL^†^	122	110.63 (45.13)
Oxidized LDL (U/L)	126	37.87 (13.46)
Triglycerides^†^	127	181.92 (119.66)
Hematocrit (%)	127	40.91 (3.72)
Iron (ng/mL)	127	80.66 (29.58)
Ferritin (ng/mL)	127	99.39 (112.11)
Transferrin^†^	127	273.10 (41.92)
Iron saturation (%)	127	20.31 (8.43)
Hepcidin (ng/mL)	126	30.46 (27.35)
Adiponectin (ng/mL)	65	8256.25 (5788.12)
TMAO (μmol/L)	127	4.59 (4.41)
RCC max (cm^2^)	124	0.35 (0.15)
RCC min (cm^2^)	124	0.28 (0.11)
RCC distensibility^‡^	125	40.69 (19.66)
RIC volume (cm^2^)	120	197.88 (60.31)
LCC volume (cm^2^)	119	177.64 (50.56)
LIC volume (cm^2^)	120	205.53 (65.39)
Total wall volume (cm^2^)	119	763.92 (182.19)

*BMI* body mass index, *BP* blood pressure, *MDRDGFR* Modification of diet in renal disease-derived glomerular filtration rate, *HDL-C* high-density lipoprotein cholesterol, *LDL-C* low-density lipoprotein cholesterol, *TMAO* trimethylamine N-oxide, *RCC* right common carotid, *RIC* right internal carotid, *LCC* left common carotid, *LIC* left internal carotid

*(mL/min/1.73 m^2^), ^†^(mg/dL), ^‡^(10^–3^/kPa)

**Fig. 1:**
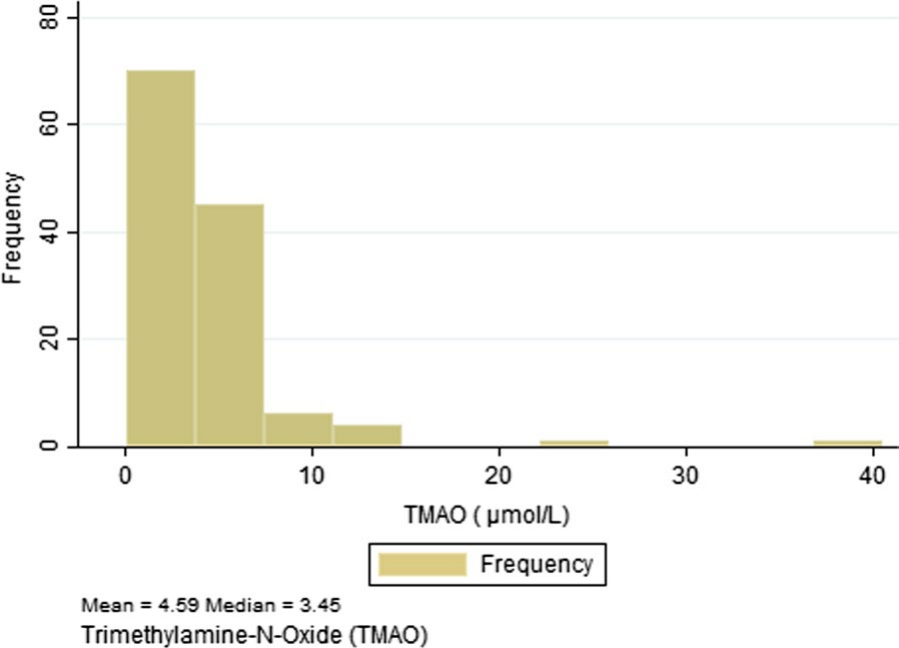
Displays the distribution of TMAO, which is right skewed with a median of 3.45 μmol/L and a mean of 4.59 μmol/L (SD = 4.4). The interquartile range of the distribution was 2.93 μmol/L (from 2.45 μmol/L, 25th percentile to 5.38 μmol/L, 75th percentile)

Figure 1 displays the distribution of TMAO, which is right skewed with a median of 3.45 μmol/L and a mean of 4.59 μmol/L (SD = 4.4). The interquartile range of the distribution was 2.93 μmol/L (from 2.45 μmol/L, 25th percentile to 5.38 μmol/L, 75th percentile).

### Ordinal logistic regression model building

Table 2 shows the results of ordinal logistic regression for the sample of 127 participants. Model 1 shows results for univariate models, Model 2 shows results for multivariable models with demographic variables age, gender and race fixed, and Model 3 shows results for the final multivariable model. Among the first two models (Models 1 and 2), statistically significant (odds ratios of *P* < 0.10) positive associations with TMAO were observed for weight (e.g. 1.01) and HDL-cholesterol (e.g. Q2 vs. Q1: 2.66, 3.01), and inverse associations were observed for transferrin (e.g. Q2 vs. Q1: 0.32, 0.29) serum iron (e.g. Q2 vs. Q1: 0.44, 0.5), iron saturation (e.g. Q2 vs. Q1: 0.57, 0.59) and total cholesterol (e.g. Q2 vs. Q1: 0.34, 0.024).

**Table 2:** Ordinal logistic regression model building

	Model 1 (Univariate)		Model 2 (Multivariable with demographics)		Model 3 (Finalized model)	
	OR (95% CI)	*P* >|z|	OR (95% CI)	*P* >|z|	OR (95%) CI)	*P* >|z|
Age	0.98 (0.89, 1.07)	0.575	–	–	0.95 (0.85, 1.05)	0.282
Gender	0.87 (0.46, 1.66)	0.674	–	–	1.10 (0.85, 1.05)	0.824
Race white versus non-white	1.29 (0.59, 2.81)	0.524	–	–	2.92 (1.02, 8.30)	0.045
Weight (lbs)	1.01 (1.00, 1.01)	0.06	1.01 (1.00, 1.01)	0.058	1.01 (0.99, 1.03)	0.251
Waist circumference	1.05 (1.00, 1.10)	0.073	1.05 (0.99, 1.10)	0.088	0.95 (0.81, 1.10)	0.491
Transferrin Q2 versus Q1	0.32 (0.13, 0.79)	0.013	0.29 (0.12, 0.72)	0.008	0.16 (0.05, 0.52)	0.002
Transferrin Q3 versus Q1	0.66 (0.28, 1.58)	0.352	0.61 (0.24, 1.51)	0.280	0.31 (0.79, 1.20)	0.090
Transferrin Q4 versus Q1	0.55 (0.23, 1.36)	0.197	0.53 (0.21, 1.30)	0.166	0.20 (0.04, 0.95)	0.043
HDL-C Q2 versus Q1	2.66 (1.07, 6.62)	0.035	3.06 (1.19, 7.85)	0.020	2.68 (0.86, 8.32)	0.088
HDL-C Q3 versus Q1	1.10 (0.46, 2.62)	0.840	1.26 (0.50, 3.19)	0.623	1.27 (0.39, 4.12)	0.687
HDL-C Q4 versus Q1	0.66 (0.27, 1.30)	0.365	0.72 (0.27, 1.85)	0.489	1.13 (0.27, 4.79)	0.868
Triglycerides Q2 versus Q1	2.02 (0.83, 4.90)	0.122	1.99 (0.81, 4.86)	0.131	2.00 (0.62, 6.48)	0.248
Triglycerides Q3 versus Q1	1.42 (0.56, 3.61)	0.464	1.40 (0.54, 3.60)	0.488	1.12 (0.31, 4.14)	0.861
Triglycerides Q4 versus Q1	2.20 (0.93, 5.25)	0.074	2.14 (0.88, 5.20)	0.091	1.68 (0.44, 6.35)	0.446
Iron Q2 versus Q1	0.44 (0.18, 1.09)	0.077	0.50 (0.19, 1.16)	0.103	1.43 (0.39, 5.25)	0.589
Iron Q3 versus Q1	0.28 (0.11, 0.68)	0.005	0.24 (0.10, 0.62)	0.003	0.55 (0.09, 3.33)	0.515
Iron Q4 versus Q1	0.63 (0.24, 1.64)	0.345	0.56 (0.21, 1.50)	0.249	2.80 (0.28, 28.26)	0.382
Saturation Q2 versus Q1	0.57 (0.24, 1.36)	0.205	0.59 (0.25, 1.42)	0.240	0.88 (0.23, 3.30)	0.845
Saturation Q3 versus Q1	0.40 (0.17, 0.96)	0.041	0.38 (0.16, 0.93)	0.033	0.44 (0.08, 2.58)	0.365
Saturation Q4 versus Q1	0.75 (0.29, 1.91)	0.543	0.68 (0.26, 1.81)	0.442	0.31 (0.03, 3.35)	0.336
Total cholesterol Q2 versus Q1	0.34 (0.13, 0.86)	0.024	0.34 (0.13, 0.87)	0.024	0.65 (0.21, 1.97)	0.444
Total cholesterol Q3 versus Q1	0.84 (0.32, 2.23)	0.726	0.84 (0.32, 2.23)	0.726	1.66 (0.52, 5.34)	0.397
Total cholesterol Q4 versus Q1	0.50 (0.20, 1.24)	0.134	0.50 (0.20, 1.24)	0.134	1.04 (0.33, 3.34)	0.944
MDRDGFR Q2 versus Q1	0.83 (0.35, 2.00)	0.679	0.89 (0.36, 2.18)	0.798		
MDRDGFR Q3 versus Q1	0.55 (0.23, 1.33)	0.184	0.58 (0.24, 1.40)	0.224		
MDRDGFR Q4 versus Q1	0.66 (0.26, 1.63)	0.361	0.70 (0.28, 1.78)	0.456		
BMI	1.03 (0.99, 1.07)	0.15	1.03 (0.99, 1.08)	0.112		
Hip circumference	1.05 (1.00, 1.11)	0.091	1.06 (1.00, 1.12)	0.056		
Systolic BP	1.01 (0.99, 1.03)	0.229	1.01 (0.99, 1.03)	0.216		
Diastolic BP	1.02 (0.99, 1.06)	0.158	1.03 (0.99, 1.06)	0.120		
LDL-C Q2 versus Q1	0.53 (0.22, 1.32)	0.174	0.55 (0.22, 1.42)	0.217		
LDL-C Q3 versus Q1	0.77 (0.31, 1.90)	0.565	0.76 (0.30, 1.90)	0.553		
LDL-C Q4 versus Q1	0.62 (0.25, 1.54)	0.305	0.66 (0.26, 1.73)	0.403		
Total volume Q2 versus Q1	1.82 (0.73, 4.55)	0.201	1.87 (0.73, 4.78)	0.190		
Total volume Q3 versus Q1	2.25 (0.91, 5.56)	0.079	2.22 (0.81, 6.06)	0.121		
Total volume Q4 versus Q1	0.96 (0.38, 2.45)	0.935	0.86 (0.26, 2.82)	0.804		
Statin use	1.43 (0.75, 2.75)	0.278	1.51 (0.76, 3.01)	0.239		
Smoking	1.46 (0.75, 2.85)	0.270	1.50 (0.75, 2.98)	0.249		
Waist-hip ratio	2.33 (0.03, 190)	0.707				
Hypertension	1.00 (0.52, 1.92)	1.00				
Diabetes	1.26 (0.65, 2.48)	0.495				
Lifetime ASCVD risk %	1.01 (0.98, 1.04)	0.726				
Creatinine	2.11 (0.40, 10.99)	0.376				
Oxidized LDL-C	1.00 (0.98, 1.30)	0.737				
Hematocrit	1.00 (0.92, 1.09)	0.932				
Ferritin	1.00 (0.99, 1.00)	0.955				
Hepcidin	1.00 (1.00, 1.02)	0.651				
Adiponectin	1.00 (0.99, 1.00)	0.485				

*HDL-C* high-density lipoprotein cholesterol, *MDRDGFR* modification of diet in renal disease-derived glomerular filtration rate, *BMI* body mass index, *BP* blood pressure, *LDL-C* low-density lipoprotein cholesterol, *ASCVD* atherosclerotic cardiovascular disease

Eight candidate variables with age, gender, and race were included in the confounder-adjusted final model (weight, waist circumference, transferrin, HDL-C, triglycerides, iron, iron saturation, and total cholesterol). Of these, only transferrin, race (white vs. non-white), and HDL-C showed significant associations (*P* < 0.10) with TMAO. Participants had 0.16 (Q2), 0.31 (Q3), and 0.20 (Q4) odds of being in a higher TMAO quartile compared with participants in the lowest transferrin quartile (Q1). Non-white participants had 2.92 times higher odds of being in the highest TMAO quartile compared to white participants. Participants in the second quartile of HDL-C had 2.68 times higher odds of being in a higher TMAO quartile compared with participants in the lowest HDL-C quartile.

**Fig. 2:**
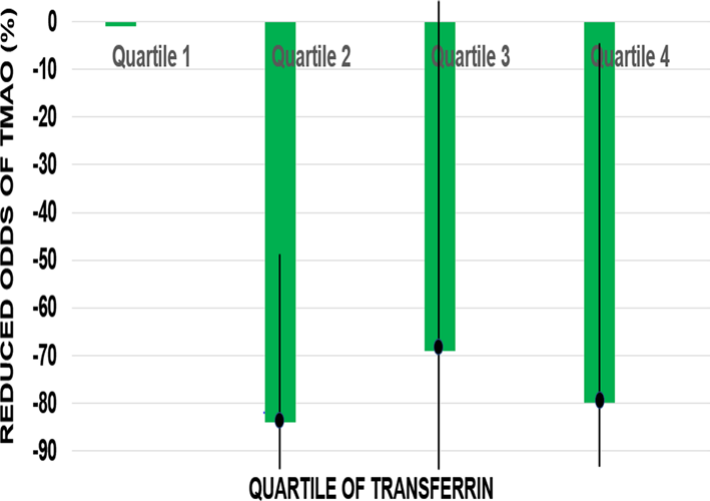
Percentage reductions in odds ratios, (1-OR) × 100%, with 95% confidence intervals, for higher versus lower TMAO levels at successively higher quartiles of transferrin. The interquartile range of transferrin was 243 mg/dL (25th percentile) to 300 mg/dL (75th percentile) with a median of 273 mg/dL (50th percentile)

The crude correlations between transferrin and TMAO was Pearson’s ρ =  − 0.0853 and Spearman’s ρ =  − 0.0645. The inverse association between TMAO and transferrin based on confounder adjusted ordinal logistic regression is better depicted in Fig. 2. The figure shows percentage reductions in the odds ratios (OR) for TMAO and transferrin from the ordinal logistic regression analysis calculated as (1-OR) × 100%. Percentage reductions in odds ratios and 95% confidence intervals for higher versus lower TMAO are plotted by quartile of transferrin. The distribution of transferrin was approximately normal with a median of 273 mg/dL (50th percentile) and interquartile range of 243 (25th percentile) to 300 mg/dL (75% percentile). For successively higher quartiles of transferrin, the odds of higher versus lower levels of TMAO was reduced by 69–84%.

## Discussion

The data used for this study had rich variety in measurements of biological, anthropometrical, dietary, demographic, and clinical variables. Because of this, the exploratory potential of this study was wide enough to make some significant observational discoveries. In our sample of 127 recruited adults 41–62 years of age with risk factors for, but without known, ASCVD, participants were selected based on having 2 or more risk factors for ASCVD (high BP, high cholesterol, DM, smoker). It is notable that the average BMI and weight was higher than a randomly selected normal population but because obesity/overweight is also a risk factor for CVD [17] and demonstrates association with other key risk factors. The prevalence of obesity in the state of Ohio, where the study was conducted, is nearly 35%, and obese individuals are likely to be overrepresented among a population interacting with the health care system, from whom this study recruited participants. It is acknowledged that the generalizability of this study is limited as this study population represents people with high risk of ASCVD and not the average physically healthy adult. In the final ordinal logistic regression, the strongest predictor variables for serum TMAO levels were race (white vs. non-white participants), transferrin, and HDL-C. An inverse association was observed between TMAO and transferrin, whereas positive associations were observed for non-white participants and HDL-C.

### TMAO and CVD

The observed inverse association of TMAO with transferrin is generally consistent with the reciprocal involvement of these factors in systemic inflammation, vis a vis., TMAO is pro-inflammatory whereas transferrin is anti-inflammatory.

Elevated TMAO concentrations have been found to induce inflammatory endothelial injury by inducing the release of inflammatory cytokines, enhancing monocyte adhesion to endothelial cells, and promoting oxidative stress [18]. Recent studies have noted that gut microbiota that synthesize TMA are iron-dependent and may thus influence iron absorption and metabolism [19].

Important clinical measures of iron absorption and delivery to tissues include serum iron and iron saturation, both of which are related to transferrin. Serum iron measures the amount of iron that is bound to transferrin in the blood, and iron saturation is the percentage of transferrin associated with iron, i.e. the percentage of total iron binding capacity. Notably, in our sample, low serum iron and low iron saturation were significant predictors of high levels of TMAO in univariate models, but these effects were attenuated in the final multivariable model after adjustment for transferrin and other variables. Furthermore, the overall mean iron saturation of our sample of participants was low (20%) suggesting that many participants may have had subclinical iron deficiency anemia or inflammatory anemia (anemia of chronic disease).

### Transferrin, TMAO and CVD

In our sample of adult participants, all of whom had CVD risk factors, transferrin was the strongest and most consistent predictor of TMAO, e.g., high levels of pro-inflammatory TMAO were markedly reduced (69–84%) in participants with higher levels of anti-inflammatory transferrin (Fig. 2). In contrast to the pro-inflammatory impact of TMAO, transferrin is a negative acute phase glycoprotein that decreases in response to inflammation. Secreted by the liver, transferrin binds to and delivers ferric iron to all tissues throughout the body. For example, transferrin-bound iron is transported to the bone marrow for the production of hemoglobin and erythrocytes.

Transferrin also chelates free iron, which is toxic, thereby preventing the formation of inflammatory free radicals and oxidative damage to the endothelium and other tissues [20]. Transferrin promotes auto-oxidation reactions which makes it vital for transporting iron in a redox-inactive form. Therefore, the primary role of transferrin is to transport iron safely around the body to supply growing cells as well as preventing free radical formation [21].

Several previous studies have reported that low transferrin is associated with systemic inflammation and increased risk of CVD. A follow up study of participants in the National Health and Nutrition Examination Survey (NHANES) reported an inverse relationship between transferrin saturation and CVD mortality [22], and a recent cohort study found that elevation of soluble transferrin receptor levels in response to iron deficiency increased the risk of myocardial infarction and cardiovascular death [23]. Other studies have also noted that the combination of anemia and inflammation (anemia of chronic disease) is associated with a worse prognosis and an increased risk of cardio-cerebrovascular death in participants with coronary artery disease [24]. These lines of evidence, coupled with our results for participants at risk for CVD, may suggest that elevated TMAO could have a part in disrupting iron homeostasis, potentially leading to inflammatory anemia that may in turn signal the presence of, if not contribute to, the pathogenesis of CVD and other CVD-related conditions. Noting that others have also suspected the role of TMAO in iron-dependent processes such as mitochondrial membrane permeability [25], this hypothesis has yet to be evaluated in an experimental design.

### Diabetes and transferrin

Several studies have linked transferrin with other chronic conditions strongly associated with CVD such as diabetes and chronic kidney disease [26–30]. As previously described, diabetes mellitus and TMAO have a well-recognized specific association. In one study, lipid peroxidation was found to be enhanced with lower concentrations of transferrin [26]. In another study, values of non-transferrin-bound iron (NTBI) levels were compared between participants with known diabetes, newly diagnosed diabetes participants, and healthy control subjects, significantly higher values of NTBI were much more common in the type 2 diabetes participants compared to the healthy controls. The highest values were observed in participants with known diabetes while the lowest were in the healthy control subjects [27]. A prospective study assessing several biomarkers of iron metabolism and insulin resistance demonstrated that serum transferrin had a statistically significant inverse association with insulin resistance [28]. Like TMAO, transferrin has been linked to diabetes indicating that diabetes may also play a role in the relationship between these two biomarkers.

### CKD and transferrin

Lower transferrin saturation has been independently associated with higher risk of mortality for CKD patients [29]. Interestingly, one study that compared hemodialysis patients with versus without CVD found that higher transferrin iron binding capacity was associated with increased CVD and end stage renal failure risk [30]. This complements our findings, as high TIBC is an indicator of iron deficiency and means that there are higher concentrations of circulating free transferrin due to low concentrations of circulating free iron to bind.

**Fig. 3:**
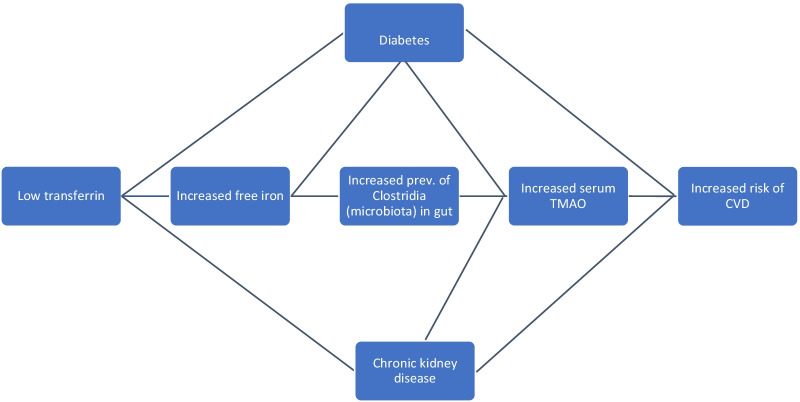
A visual diagram of the potential relationships/causal pathways between transferrin, free iron, Clostridia gut microbiota, serum TMAO, diabetes, chronic kidney disease, and CVD risk

The literature is consistent in the associations between TMAO and transferrin on chronic disease risk and outcomes, which strengthens the biological plausibility of a potential relationship between TMAO and transferrin in our study. What is clear from the discussion above is that TMAO and transferrin each demonstrate associations with various chronic diseases such as CKD, diabetes, and CVD. What remains unclear are the potential mechanistic interactions that TMAO and transferrin might have with one another. One feasible possibility is that decreased transferrin is associated with increased free iron, which favors the presence of certain gut microbiota associated with increased TMAO production [19, 31]. One study that assessed the interaction between iron availability and specific gut microbiota demonstrated that an increase in the availability of iron in the gut correlated with an increased prevalence of gut microbiota from the Clostridia class [19]. Interestingly, another study that observed the relationship between different gut microbiota and plasma metabolites such as TMAO found that there was a significant positive association between plasma TMAO concentrations and Clostridiales [31]. These two findings connect transferrin and TMAO in a biologically plausible way. The potential relationship and causal pathways of transferrin, free iron, Clostridia gut microbiota, serum TMAO, diabetes, chronic kidney disease, and CVD risk are shown as a visual diagram (Fig. 3).

### Race, TMAO and CVD

Non-white participants had 3 times higher odds of being in the highest quartile of serum TMAO compared to white participants. Similar racial differences in other CVD-related biomarkers have been reported previously, including those related to inflammation, lipids, adipokines, as well as biomarkers of endothelial damage, myocyte injury, and neurohormonal stress [32]. Our findings suggest that elevated TMAO contributes to this health disparity in CVD and CVD-related events. As a consequence of socioeconomic differences, minorities in the United States tend to have higher exposure to dietary factors that predispose them to TMAO production, inflammation and higher CVD risk [33]. Structural factors, like racism, influence the availability of healthy food choices which have been shown to be associated with CVD. As such, TMAO represents a piece of this pathway.

### Cholesterol and TMAO

It is well known that higher total cholesterol and LDL-C are associated with increased risk of CVD, whereas elevated HDL-C is generally considered protective [34]. Recent evidence suggests that atheroprotection by HDL-C is related to the enhancement of cholesterol efflux from macrophages and foam cells in atherosclerotic plaques and transport to the liver, a process known as reverse cholesterol transport [35, 36]. In this study there was a positive association between HDL-C and TMAO in certain comparisons, e.g., Q2 versus Q1. A potential biological basis for the observed association remains unclear. Presence of certain dietary interactions is a possibility, e.g. hypertriglyceridemia, hyperglycemia, and carbohydrate intake tend to be associated with lower HDL-C [37], whereas meat intake tends to be associated with higher TMAO levels. It is noteworthy that our findings contrast with those of another study in which TMAO concentration was inversely associated with HDL-C [38]. This may have been confounded by higher prevalence of diabetes and diabetic nephropathy, given the association between diabetes and low HDL-C as well as with kidney disease, which in turn is associated with higher TMAO levels.

## Conclusion

Most significantly, among participants with risk factors for (but absence of known) ASCVD, transferrin had a significant predictive association with TMAO and may represent a novel potential biomarker of increased CVD risk worthy of further study. The biological mechanistic relationships between TMAO, HDL-C, and transferrin remain unclear. These results warrant further examination of iron, metabolism, homeostasis, and gut microbiome to better understand and mitigate known increased CVD risk.

## Supplementary Information

**Additional file 1.** Dataset used for analysis.

## Data Availability

The datasets used and analyzed during the current study are available from the corresponding author on reasonable request.

## Abbreviations

TMAO: Trimethylamine-N-oxide
CVD: Cardiovascular disease
HDL-C: High-density lipoprotein cholesterol
ASCVD: Atherosclerotic cardiovascular disease
TMA: Trimethylamine
DM: Diabetes mellitus
BMI: Body-mass index
MDRDGFR: Modification of diet in renal disease-derived glomerular filtration rate
LDL-C: Density lipoprotein cholesterol
OR: Odds ratio
NHANES: National Health and Nutrition Examination Survey
NTBI: Non-transferrin-bound iron
RCC: Right common carotid
RIC: Right internal carotid
LCC: Left common carotid
LIC: Left internal carotid
